# *Staphylococcus aureus* Alpha-Toxin Mediates General and Cell Type-Specific Changes in Metabolite Concentrations of Immortalized Human Airway Epithelial Cells

**DOI:** 10.1371/journal.pone.0094818

**Published:** 2014-04-14

**Authors:** Philipp Gierok, Manuela Harms, Erik Richter, Jan-Peter Hildebrandt, Michael Lalk, Jörg Mostertz, Falko Hochgräfe

**Affiliations:** 1 Institute of Biochemistry, University of Greifswald, Greifswald, Germany; 2 Competence Center Functional Genomics, Junior Research Group Pathoproteomics, University of Greifswald, Greifswald, Germany; 3 Animal Physiology and Biochemistry, Zoological Institute and Museum, University of Greifswald, Greifswald, Germany; National Institutes of Health, United States of America

## Abstract

*Staphylococcus aureus* alpha-toxin (Hla) is a potent pore-forming cytotoxin that plays an important role in the pathogenesis of *S. aureus* infections, including pneumonia. The impact of Hla on the dynamics of the metabolome in eukaryotic host cells has not been investigated comprehensively. Using ^1^H-NMR, GC-MS and HPLC-MS, we quantified the concentrations of 51 intracellular metabolites and assessed alterations in the amount of 25 extracellular metabolites in the two human bronchial epithelial cell lines S9 and 16HBE14o^−^ under standard culture conditions and after treatment with sub-lethal amounts (2 µg/ml) of recombinant Hla (rHla) in a time-dependent manner. Treatment of cells with rHla caused substantial decreases in the concentrations of intracellular metabolites from different metabolic pathways in both cell lines, including ATP and amino acids. Concomitant increases in the extracellular concentrations were detected for various intracellular compounds, including nucleotides, glutathione disulfide and NAD^+^. Our results indicate that rHla has a major impact on the metabolome of eukaryotic cells as a consequence of direct rHla-mediated alterations in plasma membrane permeability or indirect effects mediated by cellular signalling. However, cell-specific changes also were observed. Glucose consumption and lactate production rates suggest that the glycolytic activity of S9 cells, but not of 16HBE14o^−^ cells, is increased in response to rHla. This could contribute to the observed higher level of resistance of S9 cells against rHla-induced membrane damage.

## Introduction

As a facultative pathogenic bacterium, *Staphylococcus aureus* is able to compromise the human respiratory tract [1]. Alpha-toxin, also known as alpha-hemolysin (Hla), is a major virulence factor secreted by *S. aureus* and has been recognized as an important pathogenicity determinant in *S. aureus* associated pneumonia [2]–[5]. Hla is a water-soluble protein of 33.2 kDa, which attaches to the outer surface of cells, possibly by interaction with specific plasma membrane lipids [6] or with the metalloproteinase domain-containing protein ADAM10 [7], [8]. Upon assembly of a heptameric pre-pore, Hla integrates into the membrane of host cells forming a transmembrane β-barrel pore with an inner diameter of 2.5 nm [9], [10]. In different cell types, including keratinocytes, lymphocytes and fibroblasts Hla-mediated pore-formation results in a transmembrane flux of monovalent ions and causes a drop in cellular ATP [9], [11]–[13].

Depending on the cell type, Hla can induce caspase activation and subsequent apoptosis when applied at low concentrations [14]. In contrast, high amounts of Hla trigger nonspecific integration of Hla molecules into the cell membrane which may result in necrotic cell lysis [13]. In different cell types, intracellular calcium levels are increased upon treatment of cells with Hla due to influx of Ca^2+^ ions through the plasma membrane [15], [16], but it is still unclear whether this occurs through the Hla-pore or indirectly. Although not yet directly shown, small organic molecules like ATP may pass the Hla-pore, somewhat larger molecules, however, may not, as intracellularly trapped fluorescent dye (indo-1; 650 g/mol) did not appear in the extracellular medium upon treatment of bronchial epithelial cells with 2 µg/ml Hla [15]. Likewise, a fixable dead cell-stain (Invitrogen; approximately 1,000 g/mol) applied to S9 cells after two hours pre-incubation with 0.2 µg/ml Hla did not enter the cytosol at higher rates than in untreated control cells [15].

Although mechanisms and effects of Hla pore formation as well as cellular responses to Hla treatment have been extensively studied in various cell types, including bronchial epithelial cells [7], [16]–[19], the resulting changes in cellular metabolites have not been thoroughly investigated so far. In the present work, we investigated the metabolome of the immortalized human bronchial cell lines S9 and 16HBE14o^−^. Using ^1^H-NMR spectroscopy as well as chromatographic separation coupled with mass spectrometry (GC-MS, HPLC-MS) for the detection of small molecules, we were able to define extra- and intracellular metabolic profiles for both types of cells under control conditions and at 30, 60 and 120 min after addition of a sub-lethal concentration of recombinant Hla (rHla).

## Material and Methods

### Cell culture and assay conditions

The two immortalized human airway epithelial cell lines 16HBE14o^−^ and S9 [20]–[22] are frequently used as model cells for studying cellular functions of human airways. S9 cells were originally derived from a cystic fibrosis patient, subsequently corrected by introduction of the gene encoding wild-type cystic fibrosis transmembrane conductance regulator (CFTR) through adenoviral transfer. 16HBE14o^−^ cells were derived from the bronchial epithelium of a transplant patient, express wild-type CFTR and are commonly used for analysis of a polarized cell layer [20], [23].

Both cell types were cultured in RPMI 1640 with L-glutamine (Sigma-Aldrich) supplemented with 10% heat inactivated dialyzed fetal bovine serum (FBS, Sigma-Aldrich) and antibiotics (penicillin 100 units/ml; streptomycin 100 µg/ml, Biochrom AG) at 37°C with 5% CO_2_ in a humidified atmosphere. For optimal growth, 16HBE14o^−^ cells were supplemented with L-glutamine (Biochrom AG) to a final concentration of 4 mmol/l. Medium was exchanged every third day. Cells were passaged routinely twice a week using 0.05% EDTA in phosphate buffered saline (PBS; Sigma-Aldrich) and 0.05% trypsin/0.02% EDTA in PBS (Biochrom AG). Cells were detached by gentle tapping and suspended in growth medium. An aliquot of the cell suspension obtained was mixed with trypan blue and counted using a Countess Automated Cell Counter (Invitrogen). All cultures were checked for mycoplasma contamination on a regular basis using PCR.

### Treatment with recombinant alpha-toxin

rHla was produced and purified as described [19]. For experiments, cells were seeded in 150 mm cell culture dishes (TPP Techno Plastic Products AG) in 30 ml medium at a density of 0.16×10^6^ cells/ml and left undisturbed for 24 h resulting in a confluent monolayer. Growth medium was exchanged with 10 ml of fresh medium either containing 2 µg/ml recombinant Hla or the corresponding volume of the mock purification from *Escherichia coli* containing vector DNA only. Samples were taken at 0.5, 1 or 2 h after addition of rHla.

### Sampling of medium

At each time point, 2 ml of the medium was taken and filtered through a 0.2 µm sterile filter (Sarstaedt) and directly frozen.

### Sampling of cells

Samples of cellular extracts were generated according to a modified sampling protocol reported by Teng *et al*. [24]. In brief, the supernatant was discarded and the cell culture plate was washed four times with ice-cold NaCl solution (135 mmol/l). Subsequently, 10 ml ice cold methanol was added to the plate and cells were immediately scraped from the plate and transferred into a 50 ml centrifuge tube. Next, the plate was washed with 10 ml ice cold doubly distilled water and the suspension was also transferred into the centrifuge tube. Samples were immediately frozen in liquid nitrogen.

### Extraction of metabolites

Samples were thawed on ice and 2 ml ice cold chloroform was added to each sample to perform a methanol:water:chloroform extraction with a volume ratio of 5∶5∶1 (modified after Wu *et al*. [25]). Next, the internal standards were added (20 nmol ribitol, 20 nmol norvaline and 2.5 nmol camphorsulfonic acid; Sigma-Aldrich) and the extraction solution was mixed and incubated for 10 min on ice. Subsequently, the samples were centrifuged for 10 min at 3,000×*g* at 4°C. The separated aqueous and organic phase were transferred in a new tube, mixed again and split into two samples, which were lyophilized for GC-MS analysis and for HPLC-MS analysis.

The same extraction procedure was applied to the medium samples for analyzing nucleotides in the cell supernatants. For this purpose 1.3 ml of the thawed supernatant were lyophilized. Afterwards, the samples were resuspended in 1 ml cold doubly distilled water and metabolites were extracted as described above.

### GC-MS analysis

Lyophilized samples were derivatized using a two-step method with MeOX (Sigma-Aldrich) and MSTFA (Chromatographie Service GmbH) as described [26]. For identification and quantification of amino acids, organic acids and carbohydrates a GC-MS method was used with the following setup. An Agilent Technologies GC/MS with a 7683 Series Injector, a 6890 N Network GC System and a 5973 Network Mass Selective Detector was used. An Agilent J&W GC column DB-5MS (30 m length; 0.25 mm diameter; 0.25 µm coating thickness) was applied using helium as carrier gas with constant flow of 1 ml/min. A sample volume of 2 µL was injected with a split of 1∶25. The inlet temperature was set to 250°C. The oven program was set as follows: initial temperature of 70°C was held for 1 min. Ramp 1 was set to 1.5°C/min to 76°C. Ramp 2 was set to 5°C/min to 220°C and Ramp 3 with 20°C/min to 330°C and was held for 3 min. The transfer line into the mass spectrometer was set to 280°C. Mass spectrometer settings were set as follows: MS source temperature to 230°C and the MS quadrupole to 150°C. Data acquisition rate was 20 Hz and electron ionization energy was −70 eV. The scanning mass range was between 50 and 500 amu and a solvent delay of 6 min was applied. Qualitative and quantitative analysis were performed using ChromaTOF software (LECO Corporation). Identification of peaks was carried out by comparison of mass-spectra and retention time with an in-house database. Quantification of integrated signals was performed by using a calibration from 0.5 nmol to 1000 nmol for each metabolite. Calibration curve fitting was performed with a polynomial of degree 2 and a 1/x weighting. The computed metabolite concentrations were further normalized to the sample volume and related to the respective cell number.

### HPLC-MS analysis

For identification and quantification of nucleotides and other phosphorylated metabolites a HPLC-MS with an ion-pairing reagent was used with the following setup. Chromatographic separation was performed using a RP-C_18_ AppliChrom OTU LipoMare column (3.5 µm, 150×4.6 mm) with a C_18_ phenomenex precolumn. The gradient flow rate was 0.4 ml/min and the total data acquisition runtime was 40 min. The mobile phase was A: 5% methanol and 95% water, containing 10 mmol/l tributylamine as ion-pairing reagent and 15 mmol/l acetic acid for pH adjustment to pH 4.9 and B: 100% methanol. The gradient elution started with 100% A for 2 min, 0%–31% B in 2 min, 31%–50% B in 18.5 min, 50%–60% B in 2.5 min, 60%–100% B in 1 min, hold 100% B for 7 min, 100%–0% B in 1 min, and hold 10 min at 0% B. Completely lyophilized samples were dissolved in 100 µl double distilled water and 25 µl were injected for analysis. A Bruker micrOTOF (Bruker Daltonik) mass spectrometer was operated in ESI negative mode using a mass range from 50 to 3000 m/z. Source parameters were as follows; dry gas (N_2_) flow rate: 8.0 l/min, dry temperature: 180°C, nebulizer: 1.6 bar, capillary voltage: 4000 V, capillary exit: −150 V, skimmer 1: −50 V, hexapol 1: −25 V, hexapol 2: −24 V, transfer time: 50 µs and pre-puls storage: 8.0 µs. Internal mass calibration was performed using a sodium formate solution (50% water, 50% isopropanol, 0.2% formic acid and 10 mmol/l NaOH) in negative mode for automated m/z calibration in each chromatographic run. Metabolite quantification was done by QuantAnalysis (Bruker Daltonik). Integrated signal intensities across the samples were normalized to the internal standard camphor sulfonic acid.

For determination of the calibration equation, different concentrations (0.25 nmol to 100 nmol) of pure standards were measured and analyzed in the same manner. Calibration curve fitting was performed with a polynomial of degree 2 and a 1/x weighting. The computed absolute metabolite concentrations were further normalized to the sample volumes and related to the respective cell numbers. For the extracellular metabolites measured by HPLC-MS, we only show relative signal intensities, because they were below the limit for absolute quantification.

### ^1^H-NMR analysis

400 µl of the sample was mixed with 200 µl of a sodium hydrogen phosphate buffer (0.2 mol/l, pH 7.0) including 1 mmol/l trimethylsilyl propanoic acid made up with 50% D_2_O for ^1^H-NMR analysis as described [27]. A Bruker AVANCE-II 600 NMR spectrometer operated by TOPSPIN 2.1 software was used (both from Bruker Biospin). Qualitative and quantitative data analysis was carried out by using AMIX (Bruker Biospin) as described [27].

### Statistics and data visualization

Microsoft Excel 2007 was used for all final calculations and for generation of tables. The Excel AddIn Multibase 2013 (NumericalDynamics, www.numericaldynamics.com) was used for the generation of PCA plots. Statistics and graphs were performed using Prism (version 6.01; GraphPad Software). P-values were calculated based on unpaired t-tests (after Welch) and the significance level α 0.05 was corrected by the Holm-Šidák approach to multiple comparisons (Table S1). An overview of all identified intracellular and extracellular amino acids via a hierarchical clustered heatmap was created using MeV v4.8.1 [28] with the following settings: optimized gene leaf order, euclidean distance metric and average linkage method.

### Flow cytometry

Cells (1×10^6^ per cell line) were incubated for 30 min at 4°C in a solution prepared from 5 µl PE anti-human CD156c (ADAM10) Antibody stock solution (clone SHM14; BioLegend) and 100 µl FACS buffer (PBS with 1% FBS and 3.8 mM sodium azide). Mouse IgG1 κ Isotype Control PE (eBioscience) was used as isotype control. After incubation, cells were washed twice and resuspended in 1 mL FACS buffer. Samples (10,000 events per run) were analyzed on an Attune Acoustic Focusing Cytometer (Life Technologies).

## Results

### Hla affects the cell viability of 16HBE14o^−^ cells but not of S9 cells

**Figure 1:**
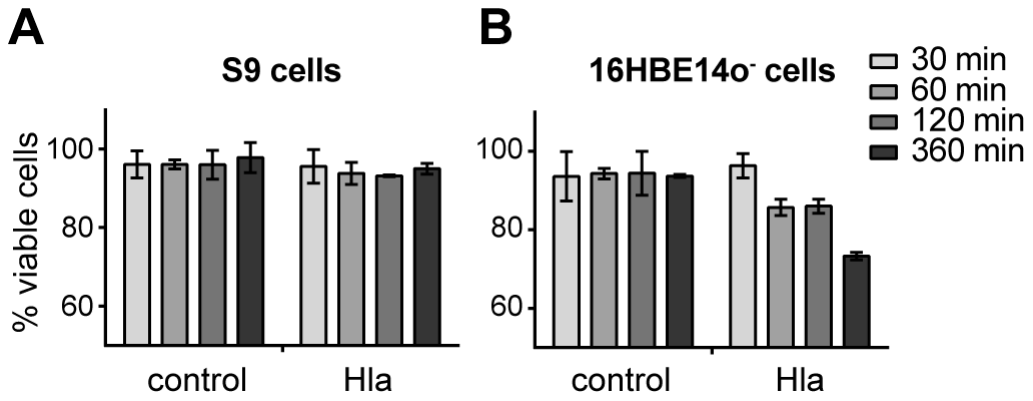
Survival of S9 and 16HBE14o^−^ cells is differentially affected by rHla treatment. Cell survival was monitored via trypan blue staining. S9 cells (**A**) and 16HBE14o^−^ cells (**B**) were counted after 30, 60, 120 and 360 min (shading from light to dark in the bar charts) without or with 2 µg/mL Hla in the cell culture medium. Living cells are presented as mean percentages of the total cell number ± SD (n = 3).

To test whether viability of S9 and 16HBE14o^−^ cells were affected by Hla, we determined the amount of living and dead cells 30, 60, 120 and 360 min after Hla exposure (Figure 1). S9 cells showed constant survival rates of greater than 90% in the presence or in the absence of 2 µg/ml rHla over 360 min. 16HBE14o^−^ cells, on the other hand, showed a distinct drop of more than 10% in viability at 60 and 120 min after addition of the toxin. The survival rates decreased by more than 20% after 360 min. These results indicate that 2 µg/mL of rHla has marked cytotoxic effects on 16HBE14o^−^ cells but no or only minor effects on S9 cells during this period of time.

To identify metabolic changes in our model cells, we therefore chose a 120 min timeframe, and profiled extra- and intracellular metabolites of S9 and 16HBE14o^−^ cells under conditions of normal growth and after application of 2 µg/ml rHla at 30, 60 and 120 min.

### Extra- and intracellular metabolic profiles of 16HBE14o^−^ and S9 cells under control culture conditions

By using ^1^H-NMR spectroscopy, altogether 25 different metabolites were identified and quantified in the cell culture medium of control cells, mainly carbohydrates and amino acids (Table S1). During the period of two hours, we detected a distinctive decrease in the concentration of glucose in the extracellular medium of S9 and 16HBE14o^−^ cells. In detail for S9 cells, the glucose concentration decreased from 11.8 mmol/l to 10.5, 10.4 and 9.6 mmol/l at 30, 60 and 120 min, respectively. For 16HBE14o^−^ cells, we detected glucose at initial concentrations of 11.2 mmol/l and 9.9 mmol/l (30 min), 8.9 mmol/l (60 min) and 7.8 mmol/l (120 min) during cultivation. Consequently, glucose levels had dropped by 2.2 mmol/l for S9 and 3.4 mmol/l for 16HBE14o^−^ cells after two hours of cultivation under control conditions.

Extracellular glutamine concentrations decreased by 365 µmol/l for S9 and 568 µmol/l for 16HBE14o^−^ cells after two hours. The concentrations of several other amino acids also declined during the culture period. Serine decreased by 74 µmol/l for S9 and 224 µmol/l for 16HBE14o^−^ cells, leucine by 111 µmol/l and 163 µmol/l, asparagine by 39 µmol/l and 29 µmol/l, lysine by 40 µmol/l and 60 µmol/l, and aspartate by 13 µmol/l and 160 µmol/l, respectively. In contrast, we observed the appearance of lactate, alanine and pyruvate which were absent in fresh culture medium. Lactate was determined at levels of 2.3 mmol/l (30 min), 2.8 mmol/l (60 min) and 4.0 mmol/l (120 min) in the cell culture supernatants of S9 cells and 2.6 mmol/l (30 min), 4.2 mmol/l (60 min) and 6.5 mmol/l (120 min) in the supernatants of the 16HBE14o^−^ cells. Likewise, alanine (100 µmol/l for S9; 196 µmol/l for 16HBE14o^−^) and pyruvate (131 µmol/l; 112 µmol/l) accumulated during the 2 h culture period under control conditions.

By using GC-MS and HPLC-MS, 51 intracellular metabolites were quantified in the intracellular metabolome, including carbohydrates, amino acids, organic acids and nucleotides (Table S1). In S9 cells, glutamate, myo-inositol, glucose, glutamine and ATP were identified as the most abundant intracellular metabolites. Likewise, major metabolites in 16HBE14o^−^ cells included glutamate, glucose, glutamine and ATP. Within the nucleotides, the nucleoside triphosphate (NTP) state was quantified with the highest absolute abundance in both model systems (Table S1). The monitored amounts after one hour of culture rank the NTPs as follows: ATP (5.96 nmol/10^6^ S9 cells; 7.77 nmol/10^6^ 16HBE14o^−^ cells), UTP (1.66; 2.27), GTP (1.33; 1.14) and CTP (0.65; 0.92). The corresponding nucleoside diphosphates (NDP) were measured in 10 to 22-fold lower amounts and rank in the same order with respect to their absolute amounts: ADP (0.37; 0.75), UDP (0.14; 0.24), GDP (0.06, 0.09), CDP (0.03, 0.06). AMP was the only monophosphorylated nucleoside we could quantify (0.02 nmol/10^6^ S9 cells; 0.04 nmol/10^6^ 16HBE14o^−^ cells after one hour) in both cell types. However, besides the monitored similarities between both model systems, differing absolute amounts of intracellular metabolites were observed in particular for myo-inositol (25.85 nmol/10^6^ S9 cells and 4.79 nmol/10^6^ 16HBE14o^−^ cells), but also for lactate (4.16 and 12.67 nmol/10^6^ cells), glutamate (25.33 and 34.14 nmol/10^6^ cells), glutamine (10.23 and 18.54 nmol/10^6^ cells) and glucose (15.23 and 11.15 nmol/10^6^ cells) one hour after fresh medium was supplied.

**Figure 2:**
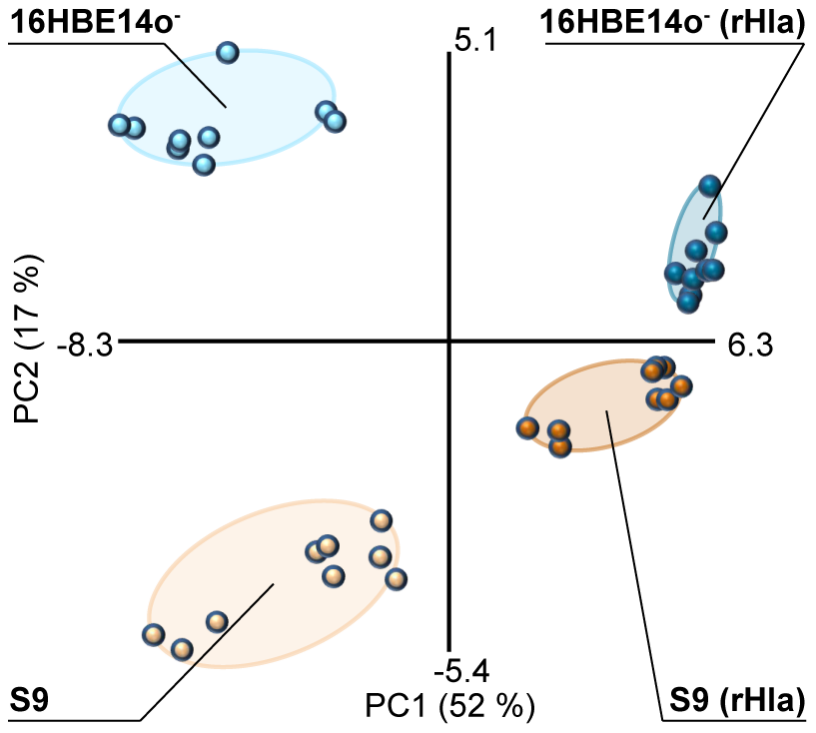
Principal component analysis of the intracellular metabolite datasets. Scores plot of the principal component analysis of the intracellular datasets of three replicates per 30, 60 and 120(light orange) and rHla-treated S9 cells (dark orange) and untreated (light blue) and rHla-treated 16HBE14o^−^ cells (dark blue).

Furthermore, marked differences became obvious for several low abundance metabolites. For example, serine (2.07 nmol/10^6^ cells), threonine (1.34 nmol/10^6^ cells) and asparagine (3.93 nmol/10^6^ cells) were found to be higher concentrated in S9 cells when compared to 16HBE14o^−^ cells (0.63; 0.66; 1.91 nmol/10^6^ cells). Finally, many tricarboxylic acid (TCA) cycle metabolites, including malate, succinate, fumarate and citrate are more abundant in 16HBE14o^−^ cells (Table S1). The intracellular metabolite data were subjected to principle component analysis that revealed a clear separation between S9 and 16HBE14o^−^ cells (Figure 2), which is mainly based on the different amounts of myo-inositol, glutamate, glutamine, lactate, glucose and asparagine.

Taken together, S9 and 16HBE14o^−^-cells show largely similar metabolic profiles and a comparable adenylate energy charge of 0.96 for S9 cells and 0.95 for 16HBE14o^−^ cells which is indicative for a high energy status of both cell types under our culture conditions. However, the observed differences in the quantities of individual metabolites point to cell type-specific metabolic features. In this context, especially the conversion rates for glucose, lactate and glutamate over two hours indicate a more than 30% higher uptake rate of glucose and glutamate in 16HBE14o^−^ cells compared with S9 cells, a difference that is mirrored by the accumulation rates of lactate in the two cell types.

### rHla enhances glucose uptake and lactate secretion in S9 but not in 16HBE14o^−^ cells

**Figure 3:**
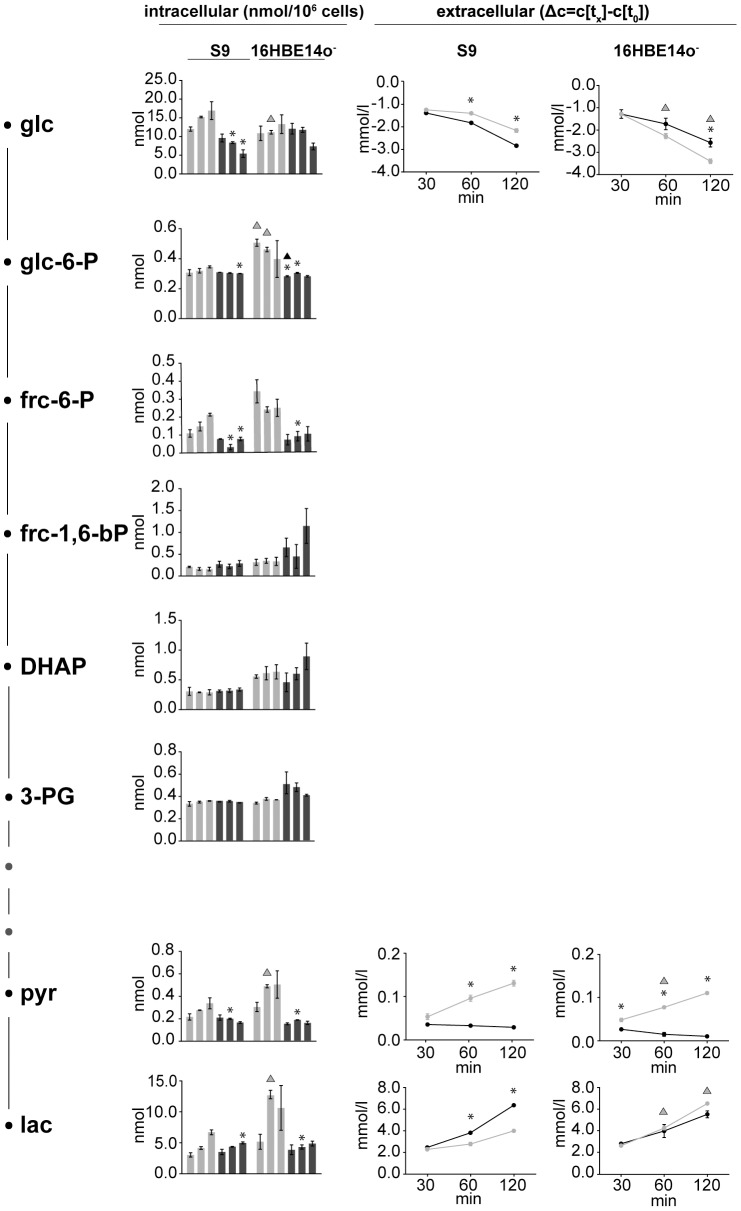
Intermediate metabolites of glycolysis. Absolute intracellular amounts of monitored metabolites of the glycolytic pathway are presented as bar charts (nmol per 10^6^ cells). Changes in the extracellular concentrations are shown as line graphs expressing the differences between the concentrations of the sample at the respective time points and the initial concentration in the medium. Data are presented as means ± SD without (light grey) and with rHla treatment (dark grey) at 30, 60 and 120 min (n = 3). Asterisks indicate significant p-values (α = 0.05 after Holm-Šidák correction) between the treated cells and the corresponding control. Triangles indicate significant p-values between the two cell lines during control conditions (grey) or during rHla-treatment (black).

To evaluate the effects of Hla on carbohydrate metabolism, we monitored intra- and extracellular metabolites involved in carbohydrate utilization and the appearance of potential end products like pyruvate or lactate after challenging the cell with rHla (Figure 3). After 30, 60 and 120 min, the concentration of glucose in the cell culture medium of rHla-treated S9 cells decreased by 1.4 mmol/l, 1.8 mmol/l and 2.8 mmol/l, respectively. Glucose consumption was hence 24% higher after 120 min in the presence of rHla when compared to S9 cells under control conditions. In contrast, glucose uptake by 16HBE14o^−^ cells was reduced during rHla treatment when compared to unstressed cells. While control cells utilized 3.4 mmol/l glucose within 120 min, only 2.6 mmol/l glucose was utilized after toxin addition. Notably, the intracellular glucose concentration was found to continuously drop in rHla-treated S9 cells compared to controls, i.e. by almost 70% within 120 min. In 16HBE14o^−^ cells, glucose was found in similar concentrations compared to control conditions at 30 and 60 min of rHla application, whereas incubation with rHla for 120 min resulted in a 45% decrease.

Lactate was observed to steadily increase in the cell culture medium of S9 cells following rHla treatment to 2.5 mmol/l after 30 min, to 3.8 mmol/l after 60 min and to 6.4 mmol/l after 120 min. Compared to control conditions, the final amount of lactate was therefore detected at an almost 1.6-fold higher level after rHla-treatment. On the contrary, less lactate accumulated in supernatants of 16HBE14o- cells during rHla treatment with levels approximately 1.0 mmol/l below the control culture after 120 min. In the same period of time, the intracellular amount of lactate, which is significantly higher in 16HBE14o^−^ cells compared with S9 cells decreases by approximately 54% and 26%, respectively.

The intracellular amounts of pyruvate, which increased somewhat during the experimental period in both types of untreated control cells, were found at constant low levels (approx. 0.16 nmol/10^6^ cells) over the experimental time period in rHla-treated cells. In contrast to the dynamics of extracellular pyruvate in control cells, pyruvate did not accumulate in the extracellular medium of rHla-treated S9 and 16HBE14o^−^ cells.

Taken together, our data indicate that Hla has severe effects on carbohydrate metabolism in a cell-specific manner. Significantly increased uptake rates of glucose by 246 nmol per hour per 10^6^ cells (p<0.001) and increased secretion of lactate by 768 nmol per hour per 10^6^ cells (p<0.001) were found in rHla-treated S9 cells, whereas lower rates compared with control conditions were observed in rHla-treated 16HBE14o^−^ cells (glucose uptake rate decreased by 169 nmol per hour per 10^6^ cells and lactate secretion by 172 nmol per hour per 10^6^ cells).

### rHla treatment increases glutamine uptake and enhances the rate of glutamine metabolism

**Figure 4:**
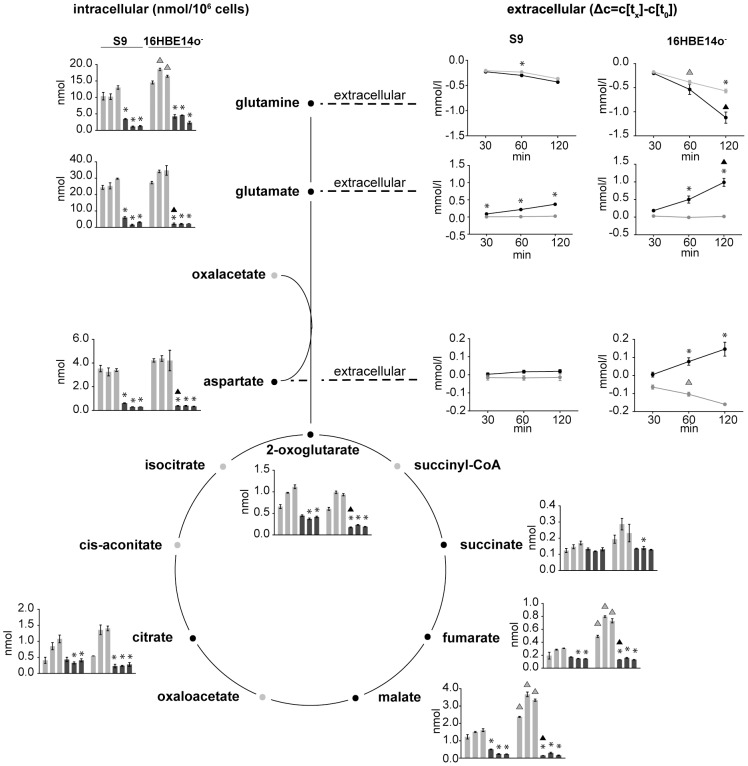
Metabolites of the TCA cycle and glutaminolysis. Schematic illustration of relevant reactions from the TCA cycle and glutaminolysis. Absolute intracellular amounts of monitored metabolites are presented as bar charts (nmol per 10^6^cells). Changes in the extracellular concentrations are shown as line graphs expressing the differences between the concentrations of the sample at the respective time points and the initial concentration in the medium. Data are presented as means ± SD without (light grey) and with rHla treatment (dark grey) at 30, 60 and 120 min (n = 3). Asterisks indicate significant p-values (α = 0.05 after Holm-Šidák correction) between the treated cells and the corresponding control. Triangles indicate significant p-values between the two cell lines during control conditions (grey) or during rHla-treatment (black).

Under control culture conditions of 16HBE14o^−^ and S9 cells, the extracellular concentrations of glutamine decreased stronger than those of any other amino acid in the cell culture medium. This indicates that these airway epithelial cells take up glutamine very efficiently. To clarify if the uptake of glutamine and pathways involved in glutamine utilization were altered during rHla treatment, we assessed concentrations of the corresponding metabolites (Figure 4). Two hours after addition of rHla to the growth medium of S9 cells, we detected a drop in the extracellular glutamine concentration by 432 µmol/l. Compared to the untreated control cells, this corresponds to an almost 20% higher glutamine uptake rate which was mediated by rHla-treatment of the cells. In 16HBE14o^−^ cells, we observed an even higher increase in glutamine uptake rate under rHla-treatment. Under control conditions, 16HBE14o^−^ cells reduced the extracellular glutamine concentration by 568 µmol/l glutamine within 2 h. After 2 h exposure to rHla, however, the cells had reduced the extracellular glutamine concentration by 1.1 mmol/l (Figure 4).

During glutaminolysis, glutamine is converted to glutamate, metabolized to 2-oxoglutarate and further distributed to diverse pathways via reactions of the TCA cycle (Figure 4). Interestingly, we detected increased amounts of glutamate in the cell culture media of S9 as well as 16HBE14o^−^ cells during rHla treatment. Glutamine and glutamate are highly abundant intracellular metabolites under control conditions in both cell lines. Addition of rHla, however, led to dramatic decreases in the intracellular contents of both metabolites (Figure 4). In general, the conversion of glutamate to 2-oxoglutarate can be coupled to the generation of aspartate from oxaloacetate. The extracellular aspartate concentration in S9 cells was only marginally affected over time under control conditions or under the influence of rHla, respectively. In the control cultures of 16HBE14o^−^ cells, a drop of 160 µmol/l in the extracellular concentration of aspartate was detected during the culture period of 2 h (Figure 4). During rHla-treatment of cells for 2 h, however, the extracellular aspartate concentration increased by 145 µmol/l. In the same time, the intracellular amount of aspartate shrunk from 3.40 to 0.31 nmol/10^6^ cells in S9 cells and from 4.21 nmol to 0.34 nmol/10^6^ cells in 16HBE14o^−^ cells (Figure 4).

The TCA cycle intermediates malate and 2-oxoglutarate were found in significantly decreased concentrations already after 30 min of rHla-application in both cell lines. In 16HBE14o^−^ cells, the amounts of malate and 2-oxoglutarate decreased to 6% and 29% of the control values, respectively. The intracellular amounts of fumarate and citrate were significantly lower in rHla-treated S9 or 16HBE14o- cells than in the respective control cells over time during rHla-treatment (Figure 4).

In summary, the data demonstrate that Hla had a strong impact on metabolites associated with glutaminolysis. Intracellular amounts of most associated metabolites declined strongly, despite an enhanced glutamine uptake by 16HBE14o^−^ cells by 145 nmol per hour per 10^6^ cells (p<0.005) and S9 cells by 29 nmol per hour per 10^6^ cells (p<0.001). Simultaneously, the extracellular glutamate concentration increased in both cell cultures, whereas the aspartate concentration increased solely in the culture supernatant of 16HBE14o^−^ cells.

### rHla mediates a decrease in the intracellular amino acid pool

**Figure 5:**
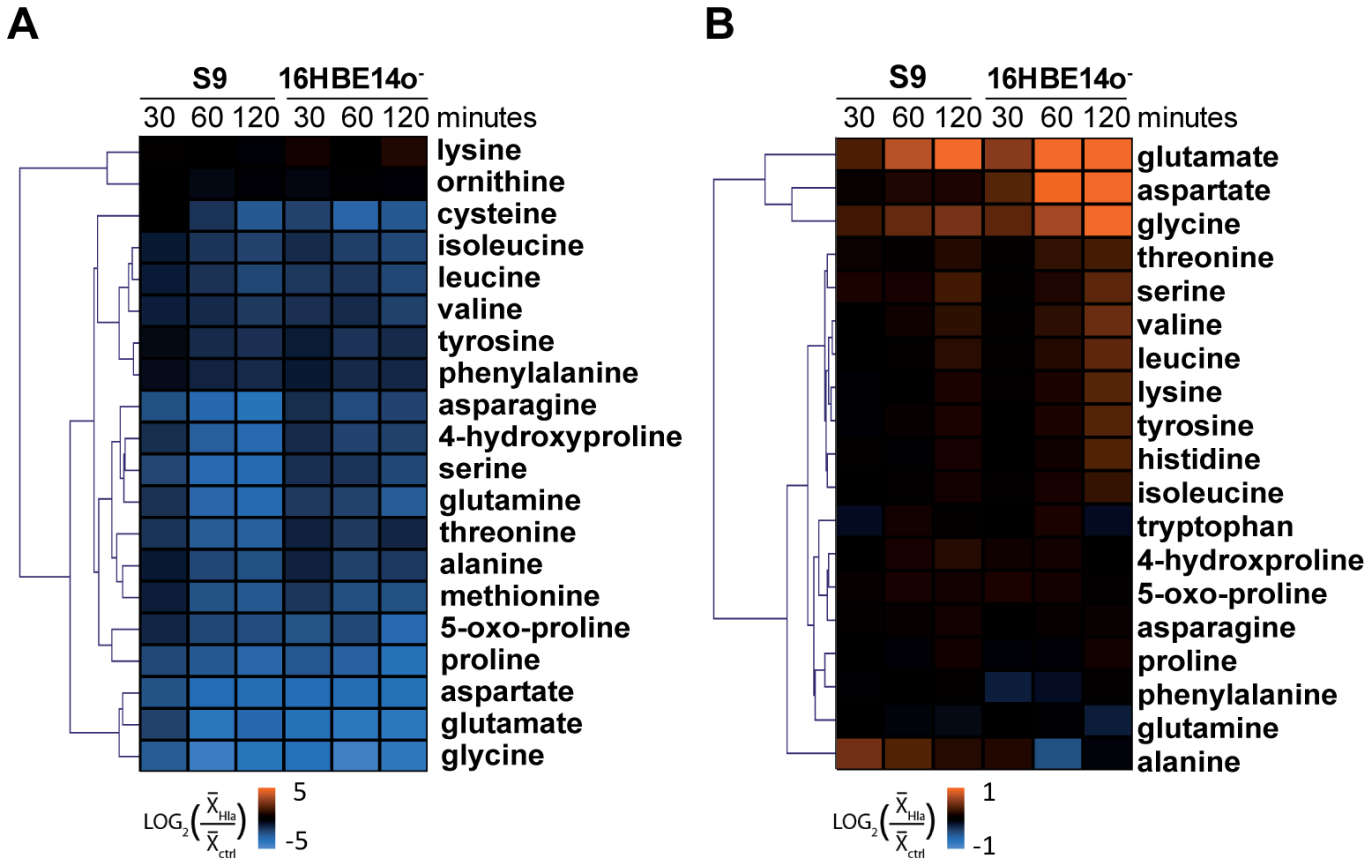
Changes in the intracellular and extracellular amino acid pool. Heatmaps of Log_2_-transformed fold changes of intra- (A) and extracellular (B) amino acids in rHla-treated vs. untreated S9 and 16HBE14o- cells. Hierarchical cluster analysis of the metabolites is based on a Euclidean distance metric.

The intracellular amounts of most of the monitored amino acids decreased in both types of cells already 30 min after the onset of exposure of cells to rHla (Figure 5A). In S9 cells, the most affected amino acids at 30 min were glycine, aspartate, asparagine, proline, serine and glutamate with decreases in concentrations between 82% – 75% compared to the respective control cells. In 16HBE14o^−^ cells, the top six affected amino acids after 30 min ranked as follows: glycine (decrease by 91%), aspartate (91%), proline (84%), 5-oxo-proline (83%) and glutamate (75%).

We also detected significant differences in the concentration time-courses of the extracellular amino acids following rHla application. In both cell types, the decline observed over time under control conditions, e.g. for threonine and serine, is attenuated during the presence of rHla in the culture medium (Figure 5B). Moreover, besides the already mentioned higher extracellular concentrations of glutamate and aspartate after rHla-treatment, glycine, which showed the steepest intracellular drop, was found in rapidly increasing concentrations in the extracellular medium of S9 as well as of 16HBE14o^−^ cells. The extracellular glycine concentration after 120 min of rHla-treatment was found to be higher in S9 cells (44%) and in 16HBE14o^−^ cells (35%) compared to the initial concentration in the medium.

### Impact of rHla on nucleotide metabolism

**Figure 6:**
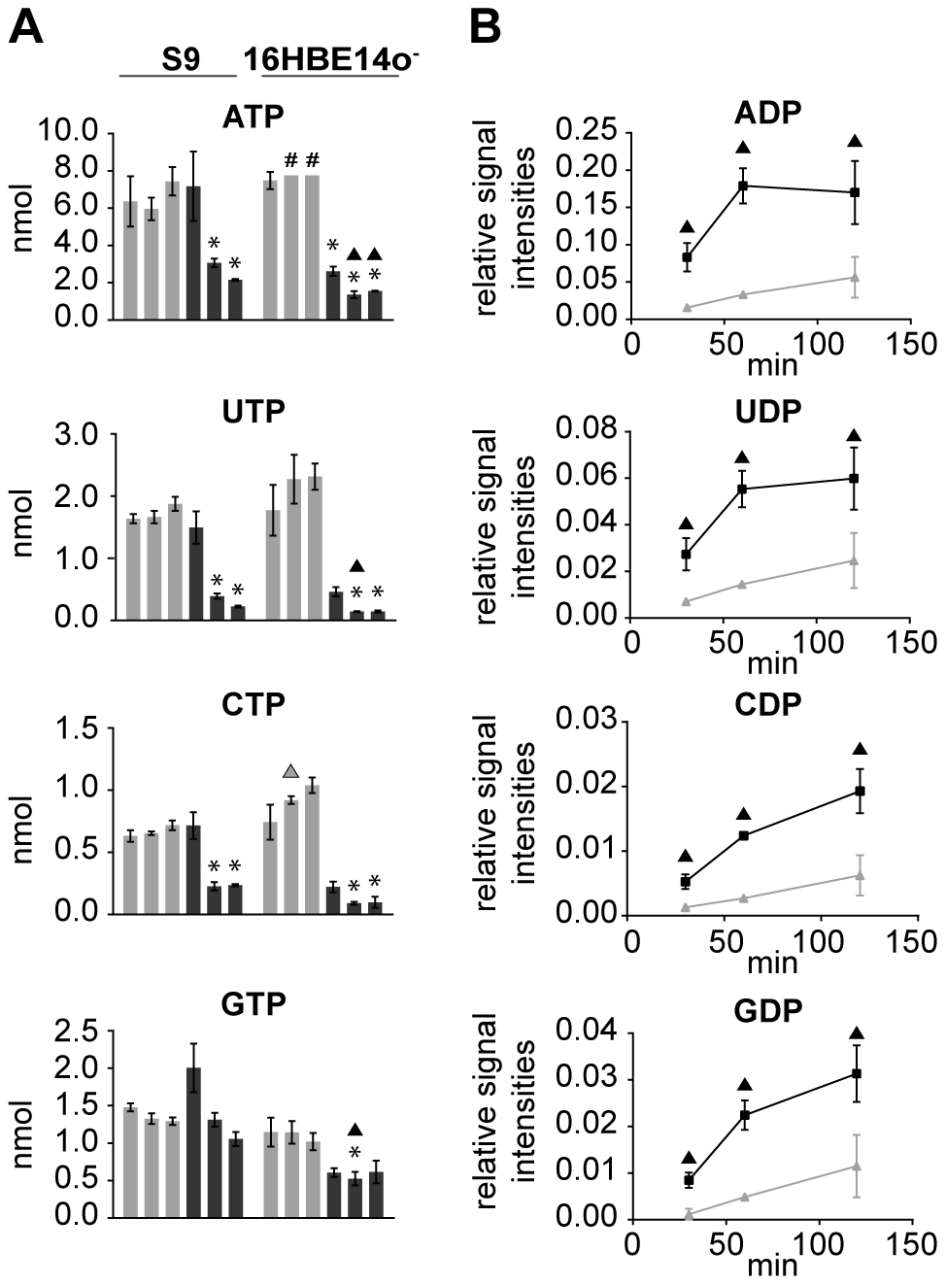
rHla-mediated changes in cellular contents and extracellular appearance of nucleoside phosphates. **A**) Absolute intracellular amounts (nmol per 10^6^ cells) of nucleoside triphosphates in S9 or 16HBE14o^−^ cells under control conditions (light grey) and during rHla treatment (dark grey) at 30, 60 and 120 min. Data are presented as means ± SD (n = 3). # indicates values that were outside the upper limit of the calibration curve and therefore set to the maximum. **B**) Changes in the extracellular levels of nucleoside diphosphates during rHla treatment as relative signal intensities (metabolite vs. internal standard) ± SD (n = 3) in the supernatants of S9 cells (light grey) and 16HBE14o^−^ cells (dark grey) at 30, 60 and 120 min. Asterisks indicate significant p-values (α = 0.05 after Holm-Šidák correction) between the treated cells and the corresponding control. Triangles indicate significant p-values between the two cell lines during control conditions (grey) or during rHla-treatment (black).

Finally, we investigated the effects of rHla on the balance of the nucleotide pool in the two bronchial epithelial cell lines using an HPLC-MS based approach. Thirty minutes after the addition of rHla, the intracellular amounts of all monitored NTPs (Figure 6A) and NDPs (Table S1) remained unchanged in S9 cells, except the amounts of GTP and GDP, which rose slightly. After 60 min of rHla treatment, ATP level decreased to 51% of the corresponding control, UTP to 23%, CTP to 24%, UDP to 35% and CDP to 41%. Two hours after addition of rHla in S9 cells, further decreases for ATP to 29%, UTP to 12%, GTP to 82%, UDP to 22% and CDP to 74% of the respective control levels were observed.

In contrast to that, in 16HBE14o^−^ cells, intracellular NTPs and NDPs were already reduced after 30 min of rHla addition to 35% of the corresponding control for ATP, 53% for GTP, 26% for UTP and 30% for CTP. After 60 min, the amounts of NTPs in 16HBE14o^−^ cells declined even further to 18% for ATP, 6% for UTP, 10% for CTP and 46% for GTP and remained at these levels up to 120 min. The GTP content, however, remained at a relatively high level of 60% of the respective control value in untreated cells at 120 min (Figure 6A and Table S1). Intracellular AMP-levels did not change much over time in 16HBE14o^−^ cells, except after 120 min of rHla-treatment at which the amount was 2.7-fold higher than in the respective controls (Table S1).

HPLC-MS analyses of the cell culture medium revealed the appearance of AMP, ADP, GDP, UDP and CDP with increasing concentrations over time for rHla-treated S9 and 16HBE14o^−^ cells which is not the case for the corresponding control cells (Figure 6B). In general, nucleotide signals for 16HBE14o^−^ cells were higher than for S9 cells. For instance, the extracellular NDPs for 16HBE14o^−^ cells are present in 4 to 7-fold higher amounts at 30 min and 60 min and approximately 2 to 3-fold higher after 120 min compared to S9 cells. As the signal intensities in general were below the lower boundary of our calibration series, we provide relative concentrations for selected nucleotides (Figure 6B).

## Discussion

The present study provides an overview of the basic metabolism of two airway epithelial cell lines, S9 and 16HBE14o^−^, and novel insights into the cell responses on a metabolic level to exposure to staphylococcal alpha-toxin (Hla). The 16HBE14o^−^ cells are highly polarized and all known characteristics indicate that these cells represent one type of serous cells at the surface of airway epithelia, whereas the less polarized S9 cells may represent an airway cell type that is derived from the lower layer of the bronchial epithelium [20], [23], [29].

Since Warburg's discovery of significant generation of lactate by cancer cells despite the presence of oxygen, the dependency on the production of energy via glycolysis and lactic acid fermentation has also been proven for fast proliferating cells and cell lines [30]–[35]. In agreement with these findings, we also observed rapid uptake of glucose simultaneously to extensive secretion of lactate even in cells under control conditions indicating that aerobic glycolysis is an important metabolic feature of S9 and 16HBE14o^−^ cells. Considering the higher rates of glucose uptake and lactate secretion in 16HBE14o^−^ cells, we presume that the glycolytic flux may be 33% higher in 16HBE14o^−^ than in S9 cells. However, calculation of the energy charge of the adenylate pool [36] revealed that the energy states of both cell types were high (approximately 0.95). During glycolysis, the consumed glucose is converted into pyruvate thereby producing ATP and NADH. Pyruvate is then reduced to lactate in order to regenerate NAD^+^ [34], [37]. Alternatively, pyruvate may also be directly secreted by the cells or it gets converted to alanine within the cells via transamination using glutamate as a nitrogen donor [34]. Since we observed increasing levels of pyruvate and alanine in the medium of cells under control conditions, both alternative pathways might play a role, although only to a minor extent considering the approximately 30 to 60-fold higher concentrations of lactate appearing in the extracellular medium. Also glutamine can be a precursor for lactate, as demonstrated for HeLa cells and transformed glioblastoma cells [34], [38]. For S9 and 16HBE14o^−^ cells, glutamine consumption rates were found to be highest of all measured amino acids. During glutaminolysis, glutamine is initially deaminated to glutamate. This step seems to occur rapidly in both cell types, because the intracellular amounts of glutamate were twice as high as the amounts of glutamine, and extracellular glutamate levels stayed constant over time. Only minor changes occurred to the intracellular amounts of glutamate indicating a constant usage of this metabolite by associated pathways such as the TCA cycle and the malate-aspartate shuttle. This is supported by the fact that we observed a rise in the intracellular TCA cycle intermediates citrate, malate and 2-oxoglutarate during the culture period of 2 h under control conditions. Although the glutaminolytic pathway may have contributed to the generation of lactate, the amount of produced lactate corresponds to 90 to 96% of the consumed glucose suggesting that most of the glucose is metabolized to lactate via aerobic glycolysis and mainly serves as energy rather than as carbon source.

We utilized the S9 and 16HBE14o^−^ airway epithelial cells to investigate the effects of staphylococcal Hla on their metabolomes. Cell viability assays revealed that 16HBE14o^−^ cells are more sensitive towards Hla-mediated cytotoxicity, whereas S9 cells are virtually resistant. Interestingly, with our metabolomics approach we could demonstrate that Hla affects the metabolic profiles at a system-wide level in both cell types. A key metabolic observation was the significant drop in intracellular ATP levels, which had also been observed when Hla was applied to human keratinocytes, fibroblasts, lymphocytes and other cell types [11]–[13], [39]. ATP may be able to leak through cell membranes of Hla-susceptible cells [40], and some authors favor the idea that it may pass directly through the toxin pores [9]. Strikingly, we observed significantly delayed decreases in the intracellular amounts of all nucleoside triphosphates as well as a delayed extracellular appearance of nucleoside diphosphates in S9 cells in comparison to 16HBE14o^−^ cells. Moreover, whereas the glucose uptake rate was lower in rHla-treated 16HBE14o^−^ cells by 20% compared to control conditions, it was increased by up to 45% (compared with cells under control conditions) in rHla-treated S9 cells and accompanied by up to 70% higher lactate production rates. Our data strongly suggest that S9 cells (in contrast to 16HBE14o- cells) are able to increase their glycolytic activity in response to rHla, thus enhancing the efficiency of their energy metabolism. This may partially counteract the loss of cellular ATP at least in the early phase of toxin action and contribute to the observed higher resistance of S9 cells against rHla-treatment.

Both cell types showed a strong decline in the intracellular amounts of glutamine and glutamate as well as extracellular glutamine concentrations upon treatment with rHla. The consumption rate of glutamine is approximately 2.3-fold higher in 16HBE14o^−^ cells compared to those in S9 cells. Extracellular glutamate, on the other hand, was found to significantly increase upon rHla-exposure in both cell types in amounts corresponding to approximately 87% of the consumed glutamine. The results indicate a strong increase in in the uptake of glutamine and subsequent hydrolysis to glutamate during rHla-treatment, especially in 16HBE14o^−^ cells. In addition, in the 16HBE14o^−^ but not in the S9 cell supernatants, aspartate accumulates in the extracellular medium. This may indicate that glutamate is further metabolized to 2-oxoglutarate which can be achieved by a coupled transamination reaction in which oxaloacetate is converted to aspartate. Since the amounts of intracellular aspartate stayed at almost constant levels during rHla-treatment of cells, we assume that aspartate is continuously produced from glutamate and released to the extracellular medium. The amount of aspartate released by 16HBE14o^−^ cells during 2 h of treatment with rHla correlates with approximately 13% of the amount of consumed glutamine and would therefore match the missing portion of the precursor glutamate that has not been released directly. The production of aspartate requires regeneration of oxaloactetate via reactions of the TCA cycle indicating ongoing activity of these reactions during rHla-treatment of cells in 16HBE14o^−^ cells. In conclusion, the results indicate that reactions of the glutaminolytic pathway are significantly more active in 16HBE14o^−^ cells than in S9 cells when rHla is present in the culture medium of the two cell types.

**Figure 7:**
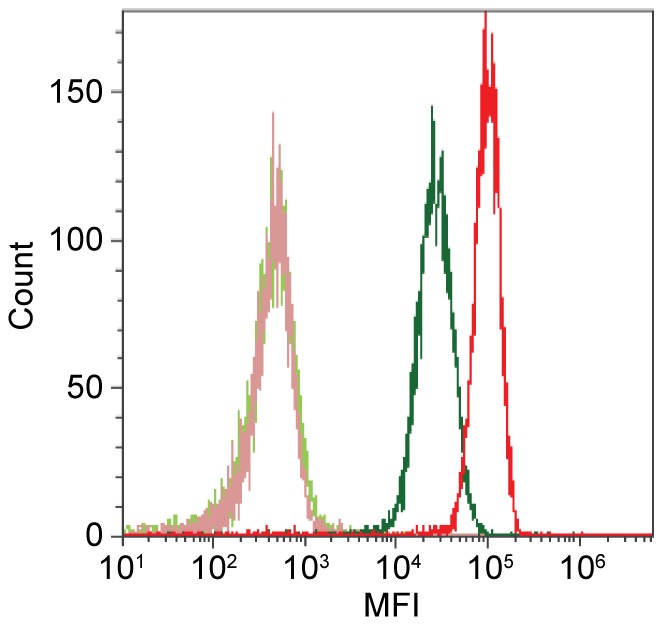
Surface expression of ADAM10. Flow cytometric analysis of ADAM10 on 16HBE14o^−^ (red line) and S9 cells (green) and corresponding negative isotype controls (light red and light green). MFI - mean fluorescence intensity.

Specific lipids and the protein ADAM10 were determined as membrane receptors for Hla [6], [7] with a probable cooperativity in the modulation of cellular responses towards the toxin [39]. Activation of ADAM10 by Hla-mediated pore formation has further been described as a major determinant of cellular injury by dissolution of cell anchorages. We have therefore undertaken analysis of the surface expression of ADAM10 with flow cytometry. Our findings indicate that 16HBE14o^−^ cells express approximately two third higher levels of ADAM10 than S9 cells (Figure 7). Although this would be in line with the observed higher sensitivity of 16HBE14o^−^ cells towards Hla-induced toxicity, it is difficult to reconcile with the finding that many metabolic changes in the distinct cells are qualitatively and even quantitatively similar. Ongoing research is needed to further dissect the interplay of cellular components involved in the mediation of the effects of Hla in terms of altered cellular responses and/or cytotoxicity.

A characteristic finding in both cell types was the drastic decline in the intracellular amounts of many organic compounds from diverse chemical classes during the presence of rHla in the culture medium, including carbohydrates, organic acids, nucleotides and amino acids. At the same time, increases in the extracellular concentrations of metabolites could be observed (Table S1). Since several of these compounds, such as nucleotides, glutathione disulfide and NAD^+^ are normally exclusively located in the intracellular space, the results may indicate that Hla mediates an increased permeability of the cell membrane to these compounds, either directly through the Hla-pore or indirectly by inducing changes in cell signaling and cell physiology inducing permeability changes of the plasma membrane. However, our results from the cell staining procedures did not indicate that plasma membrane integrity may be severely compromised in rHla-treated cells. Moreover, the ratios of changes in cellular metabolite contents and changes in extracellular concentrations were not the same for all compounds. There are different possibilities to explain these observations: 1) The Hla-pore permeabilises the plasma membranes of cells to small organic molecules and intracellular metabolites pass through the pore with different rates due to different molecular sizes, charges or other limitations. As cells may have mechanisms to remove Hla-pores from the outer membrane or to inactivate them, the rates of metabolite loss from the cytosol to the external medium may be different in 16HBE14o^−^ cells from those in S9 cells. 2) While most rHla-treated cells in a given cell type remain in a viable condition, a portion of cells may respond with loss in cell membrane integrity. Only those cells, but not the intact ones, may release their cellular content of organic molecules to the external medium. 3) Treatment of cells with rHla may induce signalling pathways which activate energy metabolism, changes metabolic pathways and secretory activities of cells which result in activation of more or less specific exit pathways for intracellular organic compounds to the external medium. Whether one or combinations of these potential explanations may be applied to the observations of this study has to be clarified in further studies.

In any case, the rHla-mediated release of a variety of intracellular substances from affected cells raises important questions about the consequences for the pathogenesis of *S. aureus*-mediated diseases in animals and in humans, such as pneumonia. The released metabolites could potentially serve as nutrients for the bacterial invader and thereby promote bacterial growth in a normally nutrient-poor or even hostile microenvironment [27]. If such a mechanism applies, bacteria could be able to thrive on the nutrients released by the host cells without killing the cells. This would enable the bacteria to survive in the host for sustained periods. On the other hand, several of the molecules found to be released following rHla-treatment are known to function as signaling molecules. The ability of extracellular nucleotides and its derivatives to modulate diverse signaling responses via purinergic receptors is well established [41], [42]. Also amino acids are important signaling molecules and play an emerging role in the regulation of immune functions [43], [44]. In this sense, release of cellular compounds may be part of the defense response of eukaryotic host cells to contact with bacterial virulence-associated factors and may help to activate mechanisms of the innate and adaptive immune responses.

To summarize our metabolomic study on the effects of rHla on the two immortalized human cell lines, S9 and 16HBE14o^−^, often used as models for human bronchial epithelial cells, the following key results seem to be important for a better understanding of the cellular consequences. The intracellular contents of many metabolites decrease during rHla-treatment of cells while some compounds appear in increasing concentrations in the external medium. Whether this reflects passive release or active secretion of individual metabolites remains to be clarified. Enhanced glycolysis, as carried out by S9 cells, stabilizes the intracellular nucleotide pool and may therefore be a critical feature for increased resistance against Hla. Enhanced glutaminolysis, as observed for 16HBE14o^−^ cells, on the other hand, is likely not sufficient to fully satisfy cellular energy needs which may provide a possible explanation for the observation that 16HBE14o- cells are more severely affected by rHla-treatment than S9 cells.

## Supporting Information

Table S1 **Metabolomics data.**
(XLSX)
